# Using EMA and Physiological Data to Explore the Relationship between Day-to-Day Occupational Stress, Musculoskeletal Pain and Mental Health among University Staff: A Study Protocol

**DOI:** 10.3390/ijerph20043526

**Published:** 2023-02-16

**Authors:** Victoria Weale, Jasmine Love, Els Clays, Jodi Oakman

**Affiliations:** 1 Centre for Ergonomics and Human Factors, Department of Public Health, La Trobe University, Bundoora, VIC 3086, Australia; 2 Judith Lumley Centre, School of Nursing and Midwifery, La Trobe University, Bundoora, VIC 3086, Australia; 3 Department of Public Health and Primary Care, Ghent University, C. Heymanslaan 10, 9000 Ghent, Belgium

**Keywords:** day-to-day work-related stress, work-related stressors, workers, musculoskeletal pain, mental health, ecological momentary assessment (EMA), physiological data, office workers

## Abstract

Exposure to work-related stressors is associated with poor physical and mental health outcomes for workers. The role of chronic stressors on health outcomes has been explored, but less is known about the potential role of exposure to day-to-day stressors on health. This paper describes the protocol for a study that aims to collect and analyze day-to-day data on work-related stressors and health outcomes. Participants will be workers engaged in predominantly sedentary work at a university. Self-report data on work-related stressors, musculoskeletal pain, and mental health will be collected three times per day for 10 work days through ecological momentary assessment via online questionnaires. These data will be combined with physiological data collected continuously via a wristband throughout the working day. The feasibility and acceptability of the protocol will be assessed via semi-structured interviews with participants and adherence to the study protocol. These data will inform the feasibility of using the protocol in a larger study to investigate the relationship between exposure to work-related stressors and health outcomes.

## 1. Introduction

Exposure to chronic work-related stressors is recognized as a growing problem, creating potentially adverse outcomes for individuals, organizations, and society, e.g., [1,2]. Adverse health outcomes from exposure to chronic work-related stressors have been reported in terms of mental health, musculoskeletal disorders, cardiovascular health, diabetes, and health risk behaviors, finding clear links between working conditions and individual health outcomes e.g., [1,3,4,5,6]. Beyond the impacts on employees’ health, work-related stressors have also been associated with negative organizational outcomes including increased turnover intentions and reduced productivity [2,7,8], and increased financial burden on society [2]. However, much of the extant research has examined exposure to chronic stressors, with limited research on acute stressors and the resultant health outcomes of such exposures [9].

Workplace stressors can arise from both physical and psychosocial working conditions; for example, undertaking physically demanding work, working to tight deadlines, having little autonomy over work, or poor relationships with colleagues and co-workers, and many jurisdictions specify that risks to psychological health must be managed [10,11]. Workers may be exposed to such risk factors on a chronic basis, or on a day-to-day basis, and irrespective of chronic or day-to-day exposure, all stressors elicit a stress response of some kind that may include physiological, affective, and behavioral responses [12], with workers’ perceptions of the work environment likely to be affected by their perceptions of how stressors are managed in the workplace.

Despite extensive research on work-related stress, significant gaps remain in terms of collecting and using data from a range of stress response dimensions, i.e., data from a combination of physiological, affective, or behavioral measures [9], limiting our understanding of the impact of stress on worker health outcomes. Standardized methods exist to measure physiological responses (e.g., heart rate, skin temperature), and a number of tools have been developed to measure the affective component, e.g., the Positive and Negative Affective Schedule (PANAS) [13]. However, whilst technology now exists to enable unobtrusive and continuous collection of physiological data (e.g., via wearable devices with sensors), affective response data may be collected less frequently (e.g., once per day). Single point-in-time date collection of affective data is problematic when aiming to identify exposure to stressors that may vary across the day and cannot be predicted. Additionally, single point-in-time measures may be subject to recall bias [14].

### 1.1. Exploring Exposure to Day-to-Day Stressors

To date, limited research investigating exposure to daily work-related stressors and outcomes has been undertaken. A recent systematic review by Lukan, Bolliger, Pauwels, Luštrek, Bacquer and Clays [9] concluded that existing research to explore day-to-day stressors has utilized a range of methodological approaches for both data collection and analysis. All studies included repeated measures; however, while most of the studies included in the review measured affective responses to stress, e.g., using the PANAS [13], only a small number of studies measured physiological outcomes and fewer examined behavioral or motivation responses. Only two included studies measured health outcomes [15,16]. Almeida and Davis [15] examined self-reported health symptoms (e.g., aches, and gastrointestinal and upper respiratory symptoms), finding workers with low flexibility to report more physical health symptoms compared to workers with high flexibility, and that a greater frequency of exposure to work stressors was associated with greater negative affect and more health symptoms for workers with low flexibility. Gervais [16] examined self-report measures of psychosomatic symptoms (e.g., backache, poor appetite, forgetfulness) among other measures (e.g., menstrual symptoms) as a predictor of job strain and job performance, finding psychosomatic symptoms to be a predictor of both higher job strain and lower job performance. Lukan et al. [9] conclude that further research to explore the relationship between day-to-day stressors and health outcomes is required, including multimodal data collection, such as via ecological momentary assessment and continuous physiological monitoring.

### 1.2. Ecological Momentary Assessment

Ecological momentary assessment (EMA) involves repeated data collection of participant behavior and experiences in real time in the natural environment [17]. As data are collected about current or recent feelings and/or experiences, EMA can overcome issues with recall, which can be a limitation of retrospective reports. Further repeated assessments allow for the assessment of variation and change in experience at different times [14]. However, some considerations are needed when using EMA. Items from existing measures may have been designed to ask participants to respond with reference to a longer timeframe (i.e., weeks, months). Consideration of whether it makes sense to ask these items in a shorter time frame is essential. Items must also be appropriate to ask several times a day. Ecological momentary assessment can place a high burden on participants. Finding a balance in the number and length of questionnaires, allowing researchers to address their aims whilst not placing too high a burden on participants, is critical.

### 1.3. Continuous Physiological Monitoring

Continuous physiological monitoring can be achieved by participants wearing relatively unobtrusive devices, such as the Empatica^®^ E4 wristband. The E4 wristband collects physiological data (skin temperature, heart rate, blood volume pulse, electrodermal activity (e.g., sweating)) as well as accelerometry. These wristbands have been used in studies exploring a range of issues; for example, seizure detection [18], atrial fibrillation episodes [19], and stress detection [20,21]. In addition to being relatively unobtrusive, another benefit of such devices is they permit the collection and visualization of real-time physiological data, enabling in-depth analysis of data collected.

### 1.4. Previous Research Including Both EMA and Continuous Physiological Monitoring

Recent research undertaken in Belgium and Slovenia (the Stress At Work (STRAW) project; see [20]) has utilized EMA to repeatedly collect data on work-related stressors, stress outcomes, health-related behaviors, and work activities, combining this with continually collected physiological data from the Empatica E4 wristband and smartphone sensor and usage data. Lukan et al. [22] reported findings from this research related to participant adherence to the study protocol and participant feedback. Lukan and colleagues [22] reported that 81% of all EMA questionnaires were fully completed, but completion varied between daytime questionnaires and evening questionnaires. Participants reported that some items were difficult to answer and found it frustrating that there was no way to return to a previous question to change an answer. On the positive side, participants liked that the questionnaires promoted self-reflection about work and experiences during the day. The EMA questionnaires were not reported to be an excessive burden, although participants reported completing fewer questionnaires on days of high work demand. The questionnaires were completed via an app on a mobile phone, and participants reported that this caused the phone battery to drain.

The protocol developed by Bolliger, Lukan, Luštrek, De Bacquer and Clays [20] was used in a recent Australian study that aimed to evaluate the feasibility and acceptability of the protocol for use in the Australian context [23]. Feasibility is concerned with whether something can be accomplished, should be accomplished, and if so, how should it be accomplished [24]. Acceptability refers to how well the protocol is received by the study population and how well it meets the needs of both the study population and the research team [25].

Talbot [23] replicated as much of the existing STRAW protocol as possible, including the use of a baseline measure and EMA questionnaires to collect data on work-related stressors, stress outcomes, health-related behaviors, and workers’ occupational activities over a 15-day period. Physiological data were also continuously collected via the Empatica E4 wristband. However, rather than collecting data from workers in universities, data were collected from workers in a private engineering firm.

Talbot [23] reported that whilst the wristband use and baseline questionnaire completion were feasible for participants, the acceptability of the baseline questionnaire was low due to the length of the questionnaire and the wording of some of the questionnaire items. Furthermore, the feasibility and acceptability of the EMA questionnaires were low due to item and scale wording, the high frequency of EMA questionnaires during the work day (approximately every 90 min), and incorrect triggering (timing) of the morning and evening EMA questionnaires. Talbot [23] concluded that whilst participant experiences were positive overall, modifications to the protocol described by Bolliger et al. [20], to increase participant feasibility and acceptability, were required for an Australian context.

The present study aims to build on the earlier research by Bolliger and colleagues [20] and Talbot [23] in a number of ways. Firstly, the protocol will be adapted to the Australian context by drawing items for the questionnaires from an existing measure that has been used in a wide range of industry settings in Australia. Secondly, the timing and frequency of questionnaires will be amended. Thirdly, EMA data will be collected via a web-based questionnaire rather than a phone app. Finally, outcomes related to musculoskeletal pain and mental health outcomes—both priority conditions to be addressed in Australian occupational health and safety strategy [26]—will be collected.

The present research aims to utilize EMA questionnaires and physiological data collected via Empatica E4 wristbands to (1) assess the feasibility of the protocol for a larger scale project; (2) analyze the relationship between occupational experiences and musculoskeletal pain; (3) analyze the relationship between occupational experiences and mental health outcomes (e.g., stress), and (4) determine the feasibility of integrating continuously collected physiological data with self-report data. This paper describes the protocol for the study. This protocol will be pilot tested to determine acceptability and feasibility of the methods.

## 2. Materials and Methods

### 2.1. Study Design

This project is a prospective observational study using data collected via wearable technology and repeated questionnaire measures. The project involves three phases. The *Preparatory Phase (Pre Day-1)* involves a 15–20 min online questionnaire to collect brief demographic and employment characteristics and data on work environment risk factors, musculoskeletal pain and mental health. The *Main Data Collection Phase (Days 1–10)* involves the continuous collection of physiological data during work hours via an E4 Empatica wristband and three daily EMAs (morning, daytime and evening) assessing work environment risk factors, musculoskeletal pain and mental health. The *Concluding Phase (Post Day 10)* involves a semi-structured interview to collect data on participant experiences with the wristband and questionnaire measures. The project has received ethical approval from the La Trobe University Human Research Ethics Committee (HEC22236).

### 2.2. Inclusion Criteria

The population of interest is university staff. Inclusion criteria are that participants need to be (1) engaged in predominantly sedentary work (e.g., sitting at a desk) at La Trobe University; (2) at least 18 years of age; (3) have access to a smartphone and a computer; (4) work at least 80% of the full-time work week in their role at La Trobe University; (5) agree to wear an Empatica E4 wristband continuously during working hours for 10 working days; (6) agree to complete the baseline questionnaire and follow-up interview and (7) have permission (verbal agreement) from their supervisor to participate in the study during work hours.

### 2.3. Participants

The study aims to recruit 20 participants, the maximum number of participants that permit data collection, taking into account equipment availability and the available data collection period. Participating for a minimum of seven out of ten working days will be considered full participation. Formal sample size calculations are deemed inappropriate for pilot studies as they are not designed to evaluate the efficacy of an intervention [27,28].

### 2.4. Recruitment

An email invitation to La Trobe University staff will be disseminated via senior department leaders; in addition, information will be posted on Yammer (the University’s internal social networking tool). The email/advert will include study aims, information about what participation involves, and eligibility criteria. Staff interested in participating will be asked to contact the research team via phone or email. The research team will check prospective participants’ eligibility, answer any questions, and enroll them in the study.

### 2.5. Procedure

In the *Preparatory Phase*, participants will be sent an invitation to the baseline questionnaire via email. Questionnaires will be administered via REDCap (Research Electronic Data Capture), a secure, web-based software platform designed to support data capture for research studies [29,30]. At the beginning of the questionnaire, participants will be asked to indicate their consent to participate. A member of the research team will schedule a visit to the participant to deliver the wristband and provide instructions on how to use it.

In the *Main Data Collection Phase*, participants will wear the wristband during work hours for 10 work days. Bolliger et al.’s protocol [20] required participants to wear the wristband and complete EMA questionnaires across 15 work days; however, in response to Talbot’s findings (see [23]), participation has been restricted to three EMA questionnaires for 10 work days in the present study. Across these 10 days, participants will be sent invitations to complete a morning, daytime, and evening EMA questionnaire. Invites will be sent via email or SMS, depending on participant preference. The timing of these surveys will be based on participants’ usual working hours: morning EMA questionnaire—one h after starting work with two reminders spaced 30 min apart; daytime EMA questionnaire—four h after starting work plus two reminders spaced 30 min apart; and the evening EMA questionnaire—one h after finishing work plus two reminders spaced one h apart. Time limits on completing the surveys are imposed to avoid overlap with the subsequent questionnaires: two h for the morning and daytime EMA questionnaires and seven h for the evening EMA questionnaire (to allow the evening EMA questionnaire to be completed prior to bedtime).

The study by Bolliger et al. [20] utilized a purpose-built app (the STRAW app) to administer EMA questionnaires; however, this is only compatible with Android mobile devices. Apple holds the majority market share of operating systems in Australia [31], so to avoid potential difficulties with recruitment, study data will be collected and managed using REDCap. Twilio, a third-party automated text messaging system integrated within REDCap [32], will be used to send SMS reminders to participants who indicate their preference is to receive invites via SMS to complete the daily EMA questionnaires. Other participants will receive the invite via email. Survey invites will contain the following message “Hi [first name]. This is the Wearables team. Please click the link for the [morning/daytime/evening] questionnaire: [link]”.

Wristbands will be worn during work hours for 10 working days. Participants will be required to transfer the data from the wristband to the E4 Connect server (a secure cloud-based server) via a computer/laptop once per day.

Following the 10 days of data collection (the *Concluding Phase*), participants will participate in a brief (20–30 min) semi-structured interview. Participants will be asked about their experiences wearing the Empatica E4 wristband and uploading data as well as their experiences completing the baseline questionnaire and daily EMA questionnaires. Notes will be taken by the interviewer during the interview and with participants’ permission interviews will be audio recorded. Interview content will be transcribed non-verbatim, and content analysis will be conducted to identify themes.

### 2.6. Measures

#### 2.6.1. Baseline Questionnaire and Ecological Momentary Assessment

Measures included in the baseline questionnaire and the morning, daytime and evening daily EMA questionnaires are presented in Table 1. Work environment risk factors will be measured using items from A Participative Hazard Identification and Risk Management toolkit (the APHIRM toolkit [33]), which has been used in a wide range of industry settings in Australia [33,34]. Items from the APHIRM toolkit [33] will also be used to measure musculoskeletal pain in the baseline questionnaire. For the daily EMA questionnaires, musculoskeletal pain items will be drawn from the Nordic Musculoskeletal Questionnaire [35].

Two scales from the Copenhagen Psychosocial Questionnaire (COPSOQ) [36], designed to measure stress and cognitive stress, will assess subjective mental health. Stressfulness will be measured using two items from the stressfulness subscale of the Stress Appraisal Measure [37]. Sleep quantity and quality will be measured using two items from the Pittsburgh Sleep Quality Index [38]. Items selected for each EMA are based on their applicability to be asked repeatedly and the relevance of the measure to the time of day (e.g., sleep items will be asked in the morning EMA questionnaire). Question stems and response options for the APHRIM items have been amended for the EMA questionnaires to suit the shorter time frame the participants will be responding in reference to.

#### 2.6.2. Physiological Measures

Physiological responses will be collected via the Empatica E4 wristband. The Empatica wristband is a wearable research device that includes well-researched and reliable stress-detection parameters including acceleration, electrodermal activity, heart rate, heart rate variability and skin temperature [39].

### 2.7. Data Management Plan

The survey data from REDCap and the physiological data from E4 Connect will be downloaded and stored on a secure password protected folder at La Trobe University that is only accessible to members of the research team named on the ethics application. Physiological data from the Empatica wristbands will be collected continuously for 10 workdays (work hours only) and manually uploaded to the E4 Connect server via the E4 Manager application, which participants will need to install on their computer. Empatica does not access personal data from study participants, the data housed in E4 Connect cannot be matched with the individuals physically wearing the device. Data will be linked to a participant code assigned by the research team.

### 2.8. Safety Considerations

No risks to health or safety of participants or the research team are identified.

### 2.9. Data Analysis

To assess the feasibility of the protocol, we will report the completion rate of the full data collection period, completion rate of the EMA questionnaires and adherence to wearing the wristband. We will use content analysis to analyze qualitative data from the semi-structured interviews to assess participant reported burden, any barriers to adherence to the study protocol, and participant reported satisfaction. To address aims 2 and 3, we will use mixed-effects models to conduct preliminary analyses of the relationships between work environment risk factors and musculoskeletal pain and mental health measured through the daily EMAs. To address aim 4, we will explore possible methods to integrate the data from the wristbands and the EMAs to assess the relationships between work environment risk factors, physiological responses and musculoskeletal and mental health outcomes.

### 2.10. Status and Timeline of the Study

The study is in the recruitment and data collection phases. Approval from the University Human Ethics Research Committee has been obtained. Recruitment materials have been prepared, and the baseline questionnaire and EMA questionnaires have been developed and tested. Recruitment commenced in early October 2022 with completion anticipated by the end of March 2023. Data collection commenced mid-October 2022, to be completed April 2023.

## 3. Discussion

This project aims to utilize wearable technology and repeated questionnaire measures via EMA to (1) assess the feasibility of the protocol for a larger scale project; (2) analyze the relationship between occupational experiences and musculoskeletal pain; (3) analyze the relationship between occupational experiences and mental health outcomes (e.g., stress), and (4) determine the feasibility of integrating continuously collected physiological data with self-reported data. This paper describes the protocol for the study. This protocol will be pilot tested to determine the acceptability and feasibility of the methods for a group of university workers. This project provides a unique opportunity to further knowledge of the impact of work-related day-to-day stressors on perceptions of musculoskeletal pain and mental health outcomes. Findings from this study will be used to assess whether a larger-scale study is feasible, but the design also enables preliminary analyses of the relationships between work-related stressors and health outcomes and between physiological data and self-report subjective data.

### Strengths and Limitations

The main strength of the present study is the combination of self-report data on work-related stressors and health outcomes collected at different time points via EMA combined with continuously collected physiological data. This approach will enable the investigation of the role of day-to-day stressors in health outcomes. The study builds on earlier work by tailoring the questionnaires used for data collection to increase acceptability in Australia, collecting data via the web rather than an app, and investigating musculoskeletal pain and mental health outcomes from exposure to work-related stressors.

A limitation of the study is the need to restrict the number of participants to 20, which is due to the availability of equipment and time constraints. However, as data are collected at multiple time points throughout the day, we believe we will have sufficient data to conduct preliminary analyses. A second limitation relates to the generalizability of the results. Participants will be employees working in predominantly sedentary roles in a university. Whilst results may be transferrable to workers in sedentary roles in other sectors, there may be some nuances specific to the tertiary education sector that influence findings. Another limitation is that selection bias may arise, in that those who are more concerned about work-related stress, or feel stressed, may be more likely to participate.

## 4. Conclusions

This study protocol describes a project that combines EMA questionnaires with continuously collected physiological data to test the feasibility and acceptability of the protocol to explore the relationship between exposure to work-related day-to-day stressors and self-reported musculoskeletal pain and mental health. This study will enable us to further knowledge and understanding of the impact of day-to-day stressors on worker health.

## Figures and Tables

**Table 1:** Study measures.

	Baseline	Morning EMA	Daytime EMA	Evening EMA
Demographic and employment characteristics				
Gender, age, living and care arrangements, educational attainment, job role, contract type, years in job, work hours, working from home status	✓	X	X	X
Physical activity and work hours	✓	X	X	✓
Work environment risk factors				
Physical environment, equipment, occupational health and safety	✓	X	X	X
Quantitative demands	✓	✓	✓	✓
Work pace	✓	✓	✓	✓
Emotional demands	✓	X	X	X
Influence at work	✓	X	✓	✓
Possibilities for development	✓	X	✓	✓
Variation of work	✓	X	✓	✓
Control over work time	✓	X	X	X
Meaning of work	✓	X	X	X
Predictability	✓	X	X	X
Recognition	✓	X	X	X
Role clarity	✓	X	X	✓
Role conflict	✓	X	X	X
Illegitimate tasks	✓	X	X	✓
Quality of leadership	✓	X	X	X
Support from supervisor	✓	X	X	✓
Social support from colleagues, sense of community at work	✓	X	X	✓
Organizational justice	✓	X	X	X
Job satisfaction	✓	X	X	✓
Work–life balance	✓	X	X	✓
Musculoskeletal pain				
Neck/shoulder	✓	✓	✓	✓
Hands, Fingers	✓	X	X	X
Arms	✓	X	X	X
Middle to lower back	✓	✓	✓	✓
Hips, bottom, legs, feet	✓	X	X	X
Mental health				
Stress	✓	✓	✓	✓
Cognitive stress	✓	✓	✓	✓
Stressfulness	X	✓	✓	✓
Sleep quantity and quality	✓	✓	X	X

## Data Availability

No new data were created or analyzed in this study. Data sharing is not applicable to this article.
